# The neurons expressing calcium-binding proteins in the amygdala of the guinea pig: precisely designed interface for sex hormones

**DOI:** 10.1007/s00429-017-1432-0

**Published:** 2017-04-29

**Authors:** Maciej Równiak

**Affiliations:** Department of Comparative Anatomy, Faculty of Biology and Biotechnology, University of Warmia and Mazury in Olsztyn, pl. Łódzki 3, 10-727 Olsztyn, Poland

**Keywords:** Amygdala, Calcium-binding proteins, Steroid hormone receptors, Immunohistochemistry, Guinea pig

## Abstract

The generation of emotional responses by the amygdala is determined largely by the balance of excitatory and inhibitory inputs to its principal neurons. These responses are often sex-specific, and any imbalance in excitatory and/or inhibitory tones leads to serious psychiatric disorders which occur with different rates in men versus women. To investigate the neural basis of sex-specific processing in the amygdala, relationships between the neurons expressing calbindin (CB), parvalbumin (PV) and calretinin (CR), which form in the amygdala main subsets of γ-aminobutyric acid (GABA)-ergic inhibitory system, and neurons endowed with oestrogen alpha (ERα), oestrogen beta (ERβ) or androgen (AR) receptors were analysed using double immunohistochemistry in male and female guinea pig subjects. The results show that in various nuclei of the amygdala in both sexes small subsets of CB neurons and substantial proportions of PV neurons co-express ERβ, while many of the CR neurons co-express ERα. Both these oestrogen-sensitive populations are strictly separated as CB and PV neurons almost never co-express ERα, while CR cells are usually devoid of ERβ. In addition, in the medial nucleus and some other neighbouring regions, there are non-overlapping subpopulations of CB and CR neurons which co-express AR. In conclusion, the localization of ERα, ERβ or AR within subsets of GABAergic interneurons across diverse amygdaloid regions suggests that steroid hormones may exert a significant influence over local neuronal activity by directly modulating inhibitory tone. The control of inhibitory tone may be one of the mechanisms whereby oestrogen and androgen could modulate amygdala processing in a sex-specific manner. Another mechanism may be thorough steroid-sensitive projection neurons, which are most probably located in the medial and central nuclei.

## Introduction

The amygdala is composed of a set of nuclei and cortical regions within the temporal lobe which are thought to be critically important for emotional behaviour and emotional learning (LeDoux 2003; Sah et al. 2003; Pape and Pare 2010), mediation of pheromonal and reproductive functions (Baum 2009; Kevetter and Winans 1981; Martel and Baum 2009; Morris et al. 2008; Segovia et al. 2006), drug addiction mechanisms (Buffalari and See 2010) and stress response (LeDoux 1994). Although the general mechanisms of all these functions are similar in both sexes, there are also sex-specific details which make these functions sexually dimorphic (Hamann 2005; Fagergren and Hurd 1999; Gruene et al. 2015; Rollins and King 2000; Swaab et al. 2003). For example, women on average retain stronger and more vivid memories of emotional events than men (Seidlitz and Diener 1998; Canli et al. 2002). In addition, the fact that emotional memories tend to be stronger in women may be linked to the greater prevalence of depression and some types of anxiety disorders in women (Bekker and van Mens-Verhulst 2007; Davidson et al. 2002). Indeed, there are many other psychological disorders involving emotional function which occur with substantially different rates in men versus women. For example, anorexia is another disorder associated with amygdala dysfunction, which is observed more often in women than in men (Swaab et al. 2003). In contrast, schizophrenia, autism and drug addiction, also associated with abnormalities in the functioning of the amygdala, are quoted more frequently in men than in women (Swaab et al. 2003). It is worth mentioning that various aspects of amygdala anatomy are sexually dimorphic (Morris et al. 2008; Równiak 2013; Równiak et al. 2015; Segovia et al. 2006).

Circulating levels of sex hormones in the bloodstream constitute additional factors which may influence amygdala physiology and may drive amygdala responses in a sex-specific manner by acting through receptors expressed in specific amygdala nuclei. Indeed, the amygdala has a high density of oestrogen and androgen receptors, as well as high aromatase expression and activity (Shughrue et al. 1997; Lephart 1996). Moreover, these receptors and aromatase were found in both the glutamatergic principal neurons (Kiss et al. 2013) and γ-aminobutyric acid (GABA)ergic interneurons (Blurton-Jones and Tuszynski 2002), suggesting that excitation and inhibition in the amygdala may be substantially modulated by sex hormones. There is a growing body of evidence that oestrogens and androgens enhance glutamatergic neurotransmission and down-regulate GABAergic neurotransmission (Barth et al. 2015; Frye et al. 2008), although opposite effects have also been reported (Tan et al. 2012; Zhou et al. 2005). For example, Murphy et al. (1998) reported that oestradiol down-regulated brain-derived neurotrophic factor (BDNF) in cultured hippocampal cells and this effect was associated with an increase in excitatory tone leading to a twofold increase in dendritic spine density and a decrease in inhibition, as evidenced by decrease in glutamate decarboxylase (GAD) and GABA level. Moreover, as both glutamatergic and GABAergic cell types express GABA receptors, an oestradiol-induced decrease in GABAergic transmission to glutamatergic cells could effectively increase excitatory drive and thus favour the formation of new dendritic spines (Murphy et al. 1998; McDonald and Mascagni 2004). Further studies have confirmed that oestradiol enhances the magnitude of long-term potentiation in the hippocampus through facilitation of the *N*-methyl-d-aspartate receptors (NMDA) (Foy 2001; Córdoba Montoya and Carrer 1997; Smith et al. 2009). On the other hand, oestrogen has an ability to alter the frequency of GABAergic miniature inhibitory postsynaptic currents (Zhou et al. 2005). Moreover, other experiments have shown that ovariectomy and subsequent oestrogen replacement can alter mRNA levels of GABA receptor subunits, GABA transporter, and GAD, the rate-limiting enzyme in GABA synthesis (Nakamura et al. 2004; Herbison et al. 1995; Herbison and Fénelon 1995).

The generation of responses by the amygdala is determined largely by the balance of excitatory and inhibitory inputs to its principal projecting neurons. The activity of these neurons is tightly controlled by GABAergic interneurons, especially these expressing calbindin (CB), parvalbumin (PV) and calretinin (CR) (Sorvari et al. 1996; Muller et al. 2007; Woodruff and Sah 2007a). It is worth mentioning that each of these subpopulations, apart from GABA, also uses different neuropeptides. For example, CB-containing neurons often co-express somatostatin (SOM) and/or neuropeptide Y, whereas CR-positive cells often co-express cholecystokinin and/or vasoactive intestinal peptide (McDonald and Mascagni 2002; Mascagni and McDonald 2003). In contrast, PV neurons are devoid of any of these neuropeptides (Mascagni et al. 2009). Moreover, it should be kept in mind that some of the CB and CR neurons are not GABAergic interneurons but glutamatergic projecting neurons (Moryś et al. 1999; Kemppainen and Pitkänen 2000; McDonald et al. 2012). If the activity of various subpopulations of GABAergic neurons was strongly influenced by circulating steroid hormones via their receptors, inhibitory control in the amygdala could easily be driven in a sex-specific manner. Such modulation may have a huge impact on the amygdala’s overall processing, and this way promotes sexually dimorphic responses. Although the amygdala has a large population of GABAergic neurons co-expressing CB, PV or CR (Pitkänen and Amaral 1994) and a large population of cells bearing oestrogen alfa (ERα), oestrogen beta (ERβ) and androgen (AR) receptors (Shughrue et al. 1997; Warembourg and Leroy 2004; Wood and Newman 1999), little is known about relationships among all these populations. There are only two studies on the relationship between neurons expressing calcium-binding proteins and steroid receptors in the amygdala, and only one of them is quantitative (Blurton-Jones and Tuszynski 2002; Perez et al. 2004). However, in both of these studies the amygdala was just one of many brain centres studied. The quantitative study of Blurton-Jones and Tuszynski (2002) describes the co-expression pattern of PV interneurons with ERβ-bearing cells in the selected nuclei of rat amygdala, where the counts were limited to the lateral and basomedial nuclei of female subjects. Perez et al. (2004) reported a lack of association between CB or PV-expressing neurons and ERα in monkey forebrain, which was also studied in female subjects only. Thus, the present study investigates for the first time the co-localization pattern of CB, PV or CR with ERα, ERβ or AR in the amygdala of the guinea pig. Female and male subjects were taken into account to examine possible sex differences as well. To make the text easier to read, the neurons expressing CB, PV, CR, SOM, GABA, ERα, ERβ and AR will be uniformly described as CB+, PV+, CR+, SOM+, GABA+, ERα+, ERβ+ and AR+ neurons.

## Materials and methods

### Subjects

Ten adult Dunkin-Hartley guinea pigs (*Cavia porcellus*, L.), five males and five females, at the age of 6 months and weighting 0.7–0.8 kg were used. All these animals were purchased from the professional supplier (Polish Mother’s Health Centre in Łódź, Poland) to ensure their proper parameters such as the same strain, the same age, similar weight and proestrus phase in female subjects. The phase of the estrous cycle was determined directly from vaginal smears. Animal care and handling were in accordance with the European Union Directive for animal experiments (2010/63/EU) and were approved by the Local Ethical Commission of the University of Warmia and Mazury in Olsztyn (No. 27/2009). All efforts were made to minimize animal suffering and to use the minimum number of animals necessary to produce reliable scientific data.

### Tissue preparation

All animals, i.e. males and females, were deeply anesthetized with an intraperitoneal injection of Morbital (Biowet, Poland; 2 ml/kg body weight, 133.3 mg/ml of pentobarbital sodium salt and 26.7 mg/ml of pentobarbital), and after cessation of breathing, immediately perfused transcardially with saline (0.9%) followed by 4% paraformaldehyde (pH 7.4) in phosphate-buffered saline (PBS). Following perfusion, brains were dissected out from the skulls, postfixed overnight in the same fixative, washed twice in 0.1 M phosphate buffer (pH = 7.4, 4 °C) and then stored for 3–5 days in graded solutions (19% and 30%) of sucrose (Sigma Aldrich) at 4 °C until they sank. Finally, the brains were frozen and then coronally sectioned at a thickness of 10 μm using a cryostat. The sections were mounted on object slides and stored at −80 °C until further processing.

### Immunofluorescence experiments

Amygdala sections were processed for routine double-immunofluorescence labelling using primary antisera raised in different species and species-specific secondary antibodies (Table 1). As each slide contained two sections, five neighbouring slides (ten neighbouring sections) were always used for staining with the use of seven different primary antibodies. Nine of these ten sections were used for the main experiment (co-localization of 3 calcium-binding proteins and 3 steroid receptors) and one spare section was used for pan-neuronal marker to visualize amygdaloid nuclei. In addition, some sections in three male and three female subjects were destined for additional staining described in the present study.

**Table 1:** Specification of reagents

Antigen	Code	Clonality	Host species	Dilution	Supplier	Location
Primary antibodies						
NEUN	MAB377	monoclonal	Mouse	1: 2000	Millipore	Temecula, CA/USA
CB	300	monoclonal	Mouse	1: 2000	SWANT	Bellinzona/Switzerland
CB	CB-38	polyclonal	Rabbit	1: 4000	SWANT	Bellinzona/Switzerland
PV	P3088	monoclonal	Mouse	1: 4000	Sigma Aldrich	St. Louis, MO/USA
CR	6B_3_	monoclonal	Mouse	1: 4000	SWANT	Bellinzona/Switzerland
ERα	06-935	polyclonal	Rabbit	1: 400	Millipore	Temecula, CA/USA
ERβ	05-824	monoclonal	Rabbit	1: 400	Millipore	Temecula, CA/USA
AR	06-680	polyclonal	Rabbit	1: 500	Millipore	Temecula, CA/USA
SOM	MAB354	monoclonal	Rat	1: 200	Millipore	Temecula, CA/USA
Secondary antibodies						
ALEXA Fluor 488	A-21202	polyclonal	Mouse	1: 800	ThermoFisher	Rockford, IL/USA
ALEXA Fluor 555	A-31572	polyclonal	Rabbit	1: 800	ThermoFisher	Rockford, IL/USA
FITC	712-095-153	polyclonal	Rat	1: 600	Jackson ImmunoLabs	West Grove, PA/USA

All samples were washed three times in PBS and then incubated for 1 h in humid chambers with blocking buffer (0.1 M PBS, 10% normal donkey serum, 0.01% bovine serum albumin, 1% Tween, 0.05% thimerosal, 0.01% NaN_3_). The sections were then rinsed in PBS and incubated overnight at room temperature with a mixture of primary antibodies, namely a combination of the appropriate primary antibody to one of the calcium-binding proteins (CB or PV or CR) and one to the steroid receptor (ERα or ERβ or AR) (Table 1). Additional incubations included neuron-specific nuclear protein NeuN (pan-neuronal marker) or mixtures consisting of CB and PV, CB and CR or SOM with one of the steroid receptors (Table 1). The antibodies were diluted in PBS containing Triton X-100 (0.3–0.5%) and 1% normal donkey serum. After incubation with the primary antibodies, the sections were rinsed in PBS and incubated for 1 h with a mixture of species-specific secondary antibodies (Table 1). Finally, all samples were rinsed in PBS and then mounted with carbonate-buffered glycerol (pH 8.6) and coverslipped.

### Controls

The specificity of the primary antisera used in this study has been shown by various researchers using these products in multiple previous studies (Airaksinen et al. 1997; Drexel et al. 2011; Meszar et al. 2012; Zimmermann and Schwaller 2002; Stanić et al. 2014; Kritzer 2004; Wood and Newman 1999). In addition, product descriptions of rabbit anti-calbindin (CB-38), mouse anti-calbindin (300), and mouse anti-calretinin (6B_3_) antisera include immunoblots of the guinea pig brain homogenates, which were specifically stained by these antibodies, showing bands at 28, 28 and 29 kDa, respectively. The same documents show also absence of specific immunohistochemical staining in brains sections of CB or CR knock-out mice using these antibodies. The rabbit antisera towards ERα (06-535), ERβ (05-824) and AR (06-680) were tested by immunoblots and these antibodies specifically stained the mouse brain extracts showing single bands at 66, 59 and 110 kDa, respectively (Stanić et al. 2014). The specificity of secondary antibodies was controlled by the omission and replacement of all primary antisera by non-immune sera or PBS. Lack of any immunoreactions indicated specificity.

### Counts and statistics

Colocalizations of CB+, PV+ and CR+ cells with particular steroid receptors were analysed in the lateral, basolateral, basomedial, central, medial and cortical amygdaloid nuclei using an Olympus BX51 microscope equipped with Cell-F image analysis software (Olympus GmbH, Germany). Only the numbers of single- and double-labelled CB+, PV+ and CR+ cells were counted. Single-labelled ERα+, ERβ+ or AR+ cells were excluded from the investigation. For each nucleus in each animal of both sexes, particular combinations of antigens were counted on 10 evenly spaced sections arranged from the rostral to the caudal extent. The space between sections analysed per specific nucleus depended on the nucleus length, and these values were as follows: LA: 250 µm, BL: 200 µm, BM: 200 µm, CE: 200 µm, ME: 200 µm and CO: 250 µm. In order to confirm the localization of the individual nuclei on the sections, neighbouring sections stained with mouse anti-NeuN (pan-neuronal marker) were used. All counts on the single section were made at 20× magnification using 347.6 μm × 260.7 μm regions. The test frames were arranged in a way that ensured coverage of the whole cross-sectional area of each nucleus studied. The numbers of test frames studied per nucleus were as follows: LA: 5–7, BL: 2–4, BM: 2–3, CE: 2–4, ME: 3–4 and CO: 2–3. Within test frames single-labelled and double-labelled neurons were counted separately. Such separate counts made within the test frames in the single nucleus in the subject were totalled. Finally, counts from each nucleus were averaged in each sex group and expressed as mean ± standard deviation (SD). All counts were made on coded slides by the author. To avoid fluorescence fading, test frame was digitally recorded before counting. Such digital frames were in the form of stacks which were composed of two microphotographs representing red and green immunofluorescence channels. Saved stacks were also evaluated by two independent experimenters, being blind to the parameters of the studied tissues (sex, nucleus, antigens, etc.). The results of these counts showed high inter-rater reliability (Pearson *R* = 0.86, *p* < 0.001). Statistical differences between the two experimental groups (males vs. females) were analysed with a two-tailed *t* test (*p ≤ 0.05, **p ≤ 0.01 and ***p ≤ 0.001) using GraphPad Prism 5 software (GraphPad Software, La Jolla, CA, USA).

As the population of CB+ neurons in the amygdala consists of two separate and non-overlapping subpopulations such as CB+/PV+ and CB+/SOM+ cells, additional staining was performed to clarify the relationships between CB+ neurons and either PV+ or CR+ cells, as well as between SOM+ neurons and either ERβ+ or AR+ cells. All these sections were analysed using an epifluorescence microscope. However, the SOM signal was present only in cytoplasm, while the AR signal was present only in cell nuclei, and thus in many double-stained cells the colocalization was not obvious. Therefore, to unequivocally ascertain that SOM and AR co-exist in the same neurons, additional analysis was performed with the use of a confocal microscope. All additional sections were also evaluated quantitatively in a way described above (5 sections per nucleus in 3 male and 3 female subjects). In the case of CB staining, only single and double-labelled CB+ neurons were counted, whereas single-labelled PV+ or CR+ cells were excluded from analysis. In the case of SOM preparations, only single- and double-labelled SOM+ neurons were counted, while single-labelled ERβ+ or AR+ cells were excluded from investigation.

### Photomicrographic production

Low- and high-magnification photomicrographs of immunoreactive cells were taken with a CC-12 digital camera (Soft Imaging System GMBH, Germany) on an Olympus BX51 microscope. These digital images were slightly modified to optimise the image resolution, brightness and contrast using CS4, version 11.0, software (Adobe Systems Inc., San Jose, CA, USA). Low- and high-magnification confocal fluorescent images (Fig. 4d–f) were obtained with a Leica TCS SP5 microscope (Wetzlar, Germany). Sequential excitation at 488 nm and 561 nm was provided by argon and DPSS 561 lasers, respectively. Schematic drawings demonstrating the distribution patterns of ERα+, ERβ+ and AR+ cells were created by marking with a dot each neuron on the digitalized section and superimposing outlines from the adjacent anti-NeuN sections.

## Results

### Distribution of CB+, PV+, CR+ and SOM+ neurons in the amygdala of male and female guinea pig

The position of the various nuclei in the guinea pig amygdala as well as the distributions of CB+, PV+ and CR+ neurons in this region were precisely described in both sexes in our previous reports concerned with sex differences existing in this species (Równiak 2013; Równiak et al. 2015). The distribution pattern of SOM+ neurons in the guinea pig amygdala was very similar to that described in the rat (McDonald 1989) and pig (Równiak et al. 2008). In the present study, the same six main amygdaloid regions, i.e. the lateral, basolateral, basomedial, central, medial and cortical nuclei were chosen for detailed investigation. In all these regions, the relationships between CB+, PV+ or CR+ neurons and ERα+, ERβ+ or AR+ cells were analysed and compared between the sexes.

### Distribution of ERα+, ERβ+ and AR+ neurons in the amygdala of male and female guinea pig

**Fig. 1:**
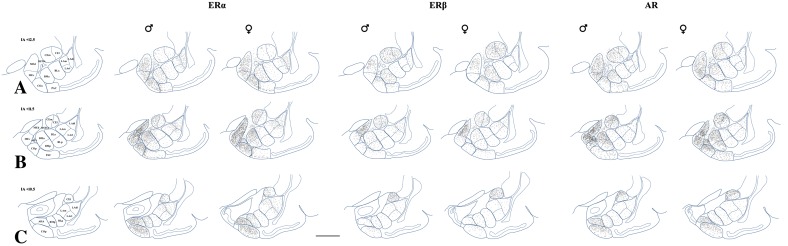
Computer-generated plots illustrating an overview of the distribution of ERα+, ERβ+ and AR+ neurons in the whole amygdala at various coronal levels in the male and female subjects. Note the differences in the density of ERα+, ERβ+ and AR+ cells at various coronal levels. Note also differences in the density of ERα+, ERβ+ and AR+ cells between “cortex-like” and “striatum-pallidum-like nuclei”. *Each dot* represents one immunostained nucleus. Three coronal levels are presented (**A** is the most rostral, **B** is middle, and **C** is the most caudal). The numbers shown in the upper left corners represent distances in millimetres from the interaural plane (IA) which was used as a rostrocaudal zero reference point for the coronal sections in the guinea pig forebrain atlas of Tindal (1965). These stereotaxic coordinates are used only as a guide, and are given to allow the reader to refer to the atlas. *Scale bar* 2000 µm in **C** (applies to all sections). *AHA* amygdalohippocampal area, *BLa* basolateral nucleus, anterior part, *BLp* basolateral nucleus, posterior part, *BMa* basomedial nucleus, anterior part, *BMp* basomedial nucleus, posterior part, *BSTiA* bed nucleus of the stria terminalis, intraamygdaloid part, *CEl* central nucleus, lateral part, *CEm* central nucleus, medial part, *COa* anterior cortical nucleus, *COp* posterior cortical nucleus, *I* intercalated nucleus, *LAdl* lateral nucleus, dorsolateral part, *LAvl* lateral nucleus, ventrolateral part, *LAm* lateral nucleus, medial part, *MEd* medial nucleus, dorsal part, *MEv* medial nucleus, ventral part, *PAC* periamygdalod cortex

**Fig. 2:**
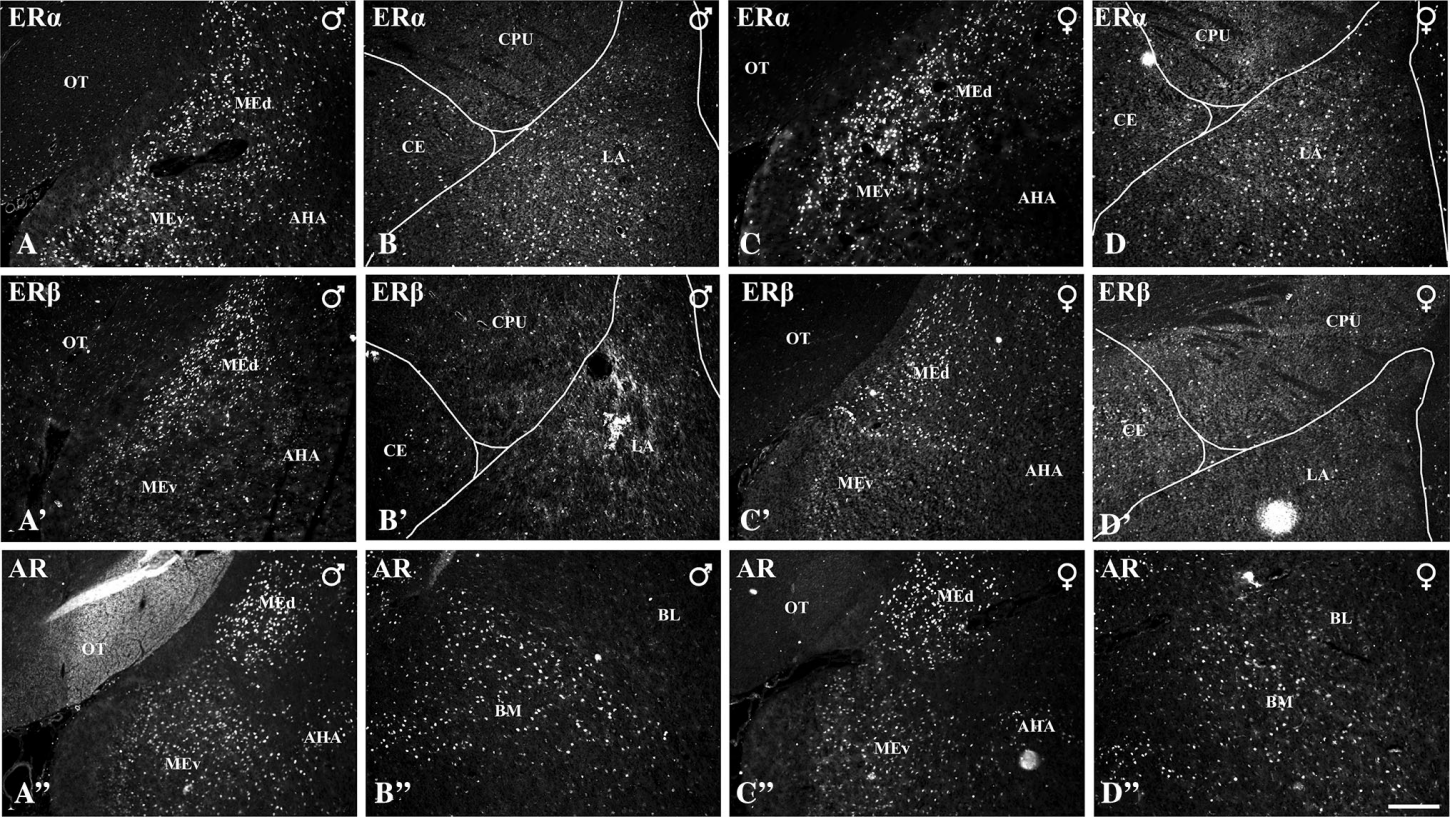
Distribution of neurons expressing ERα (**A**–**D)**, ERβ (**A’**–**D’**) or AR (**A”**–**D”**) within the amygdala of male (**A**–**B**, **A’**–**B’**, **A”**–**B”**) and female (**C**–**D**, **C’**–**D’**, **C”**–**D”**) guinea pigs. **A**–**D**: low-magnification view of the ERα immunofluorescence showing large numbers of ERα+ neurons in the medial and lateral nuclei. **A’**–**D’**: low-magnification view of the ERβ+ immunoreactivity showing large numbers of ERβ+ cells in the medial but not lateral nucleus. **A”**–**D”**: low-magnification view of the AR+ immunofluorescence demonstrating densely arranged AR+ cells in the medial and basomedial nuclei. Note that within the medial nucleus ERα+ neurons are densely arranged in both parts, ERβ+ cells are more numerous in dorsal part and AR+ neurons are more intensely stained in the dorsal part. *Scale bar* 200 µm in **D”** (applies to all sections). *AHA* amygdalohippocampal area, *BL* basolateral nucleus, *BM* basomedial nucleus, *CE* central nucleus, *CPU* caudatoputamen, *LA* lateral nucleus, *ME* medial nucleus, *MEd* dorsal part, *MEv* ventral part, *OT* optic tract

An analysis of the distributions of ERα+, ERβ+ and AR+ immunoreactivities in the amygdala of male and female guinea pig revealed large numbers of weakly to strongly stained cells throughout the entire rostral to caudal continuum of the amygdala (Figs. 1, 2). In both sexes, ERα+ cells were the most numerous, while ERβ+ neurons were the least numerous. The subcellular distribution of these immunoreactivities was predominantly nuclear in most neurons (Fig. 2). However, in some cells, the cytoplasm and neuronal processes were also immunostained.

Although, populations of ERα+, ERβ+ and AR+ neurons had slightly different patterns of distribution in the amygdala, these three patterns also had many common features (Figs. 1, 2). For example, in both sexes, the ERα+, ERβ+ and AR+ cells were the most numerous and the most densely packed in the medial nucleus (Figs. 1, 2). Moreover, in the posterodorsal portion of the medial nucleus, the ERα+, ERβ+ and AR+ cells were among the most intensely labelled, whereas in the other nuclei of the amygdala the intensity was weaker. Another characteristic and common feature was clusters of intensely stained ERα+, ERβ+ and AR+ cells in the intra-amygdaloid division of the bed nucleus of the stria terminalis and scattered neurons in the central nucleus. In the basolateral complex of the amygdala, only the basomedial nucleus contained many ERα+, ERβ+ and AR+ cells. On the other hand, in the lateral and basolateral nuclei fewer ERα+, ERβ+ and AR+ signals were found (Figs. 1, 2). However, in the lateral nucleus, the density of ERα+ nuclei was quite moderate. In the amygdalohippocampal area, ERα+ and AR+ cells were abundant, but relatively few ERβ+ cells were found (Figs. 1, 2). In contrast, ERα+ cells were much more numerous than ERβ+ or AR+ neurons in cortical nuclei (Figs. 1, 2).

### Co-existence pattern of CB+, PV+ and CR+ neurons with ERα, ERβ or AR in the amygdala of male and female guinea pig

**Fig. 3:**
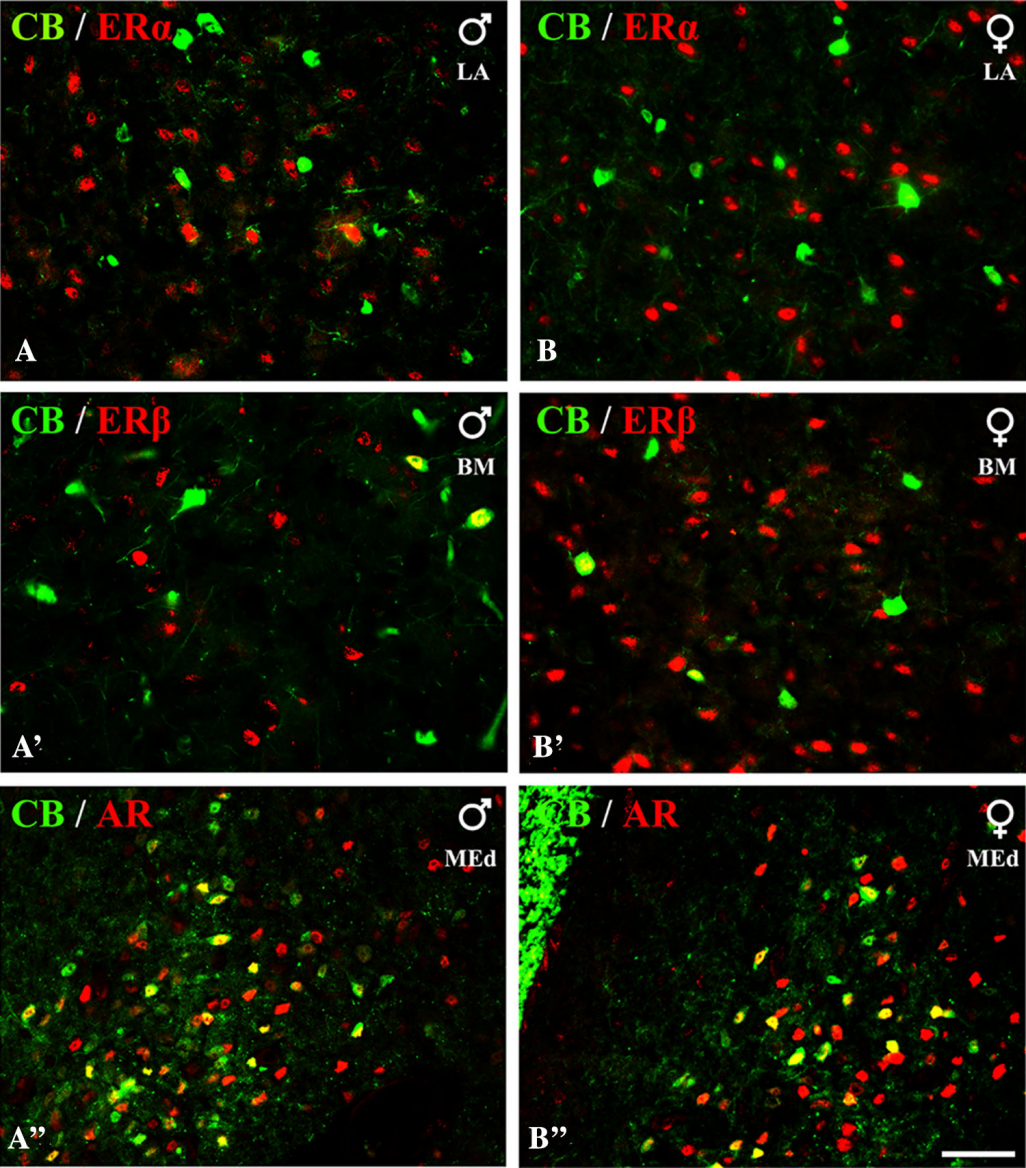
Representative colour photomicrographs illustrating the anatomical relationships between CB+ neurons and nuclei immunoreactive for ERα (**A**, **B)**, ERβ (**A’**, **B’**) or AR (**A”**, **B”**) in the amygdala of male (**A**, **A’**, **A”**) and female (**B**, **B’**, **B”**) guinea pigs. Note, that a part of neurons that were immunopositive for CB were also ERβ+ (**A’**, **B’**) or AR+ (**A”**, **B”**), but virtually none of them were ERα+ (**A**, **B)**. *LA* lateral nucleus, *BM* basomedial nucleus, *MEd* dorsal part of medial nucleus. *Scale bar* 50 µm in **B”** (applies to all sections)

**Fig. 4:**
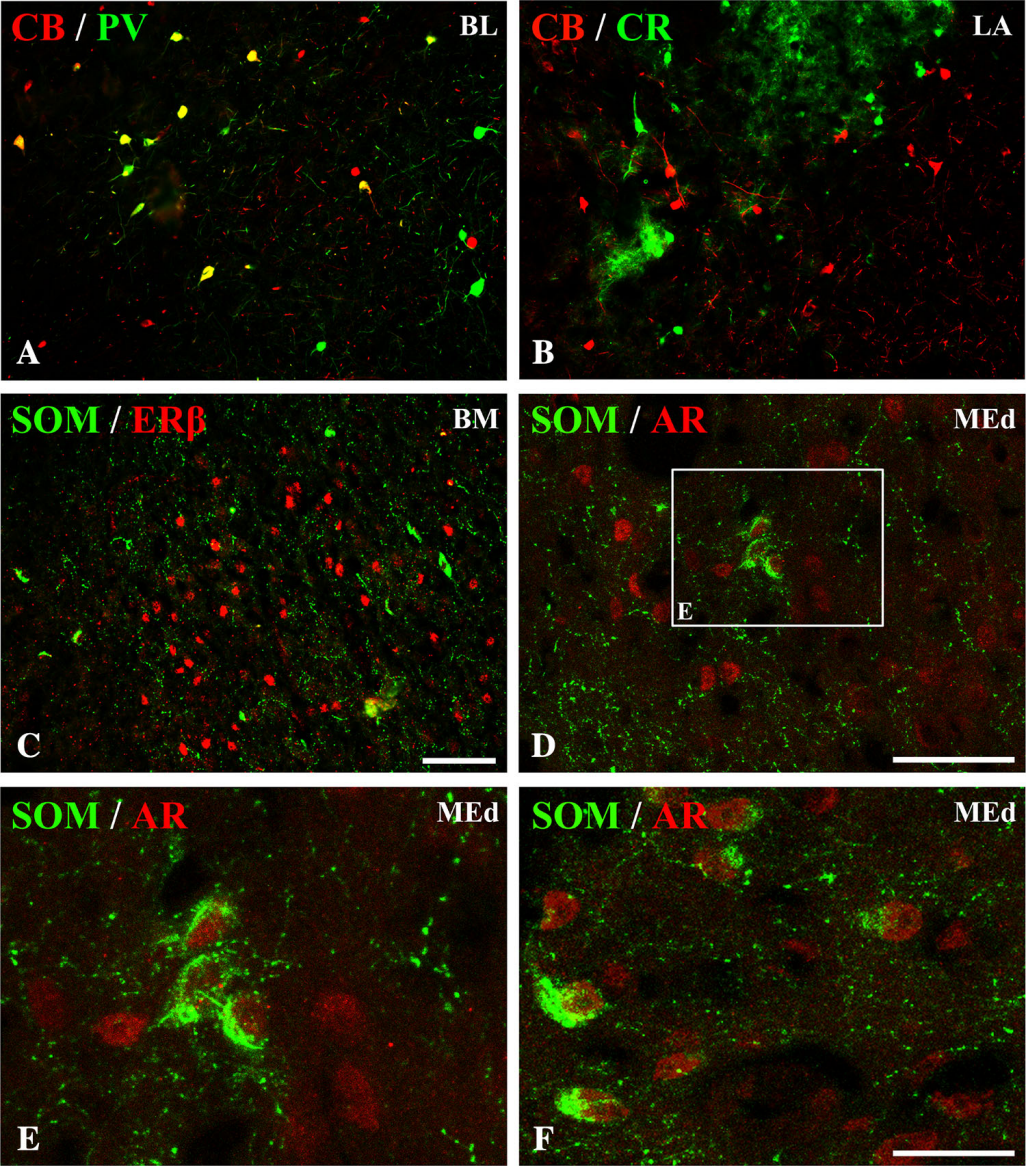
Representative colour photomicrographs illustrating the anatomical relationships between neurons expressing CB and PV (**a**), CB and CR (**b**), SOM and ERβ (**c**) and SOM and AR (**d**–**f**) in the amygdala in the guinea pig. *Boxed* region in **d** is shown at higher magnification in **e**. Note, extensive co-localization of CB with PV (**a**) and the lack of co-expression of CB with CR in the same neurons (**b**). Note, also that SOM and ERβ do not co-exist in the same neurons (**c**) while SOM+ neurons were often simultaneously AR+ (**d**–**f**). *BL* basolateral nucleus, *LA* lateral nucleus, *BM* basomedial nucleus, *MEd* dorsal part of medial nucleus. *Scale bar* 100 µm (**a**–**c**), 75 µm (**d**) and 25 µm (**e**–**f**)

**Fig. 5:**
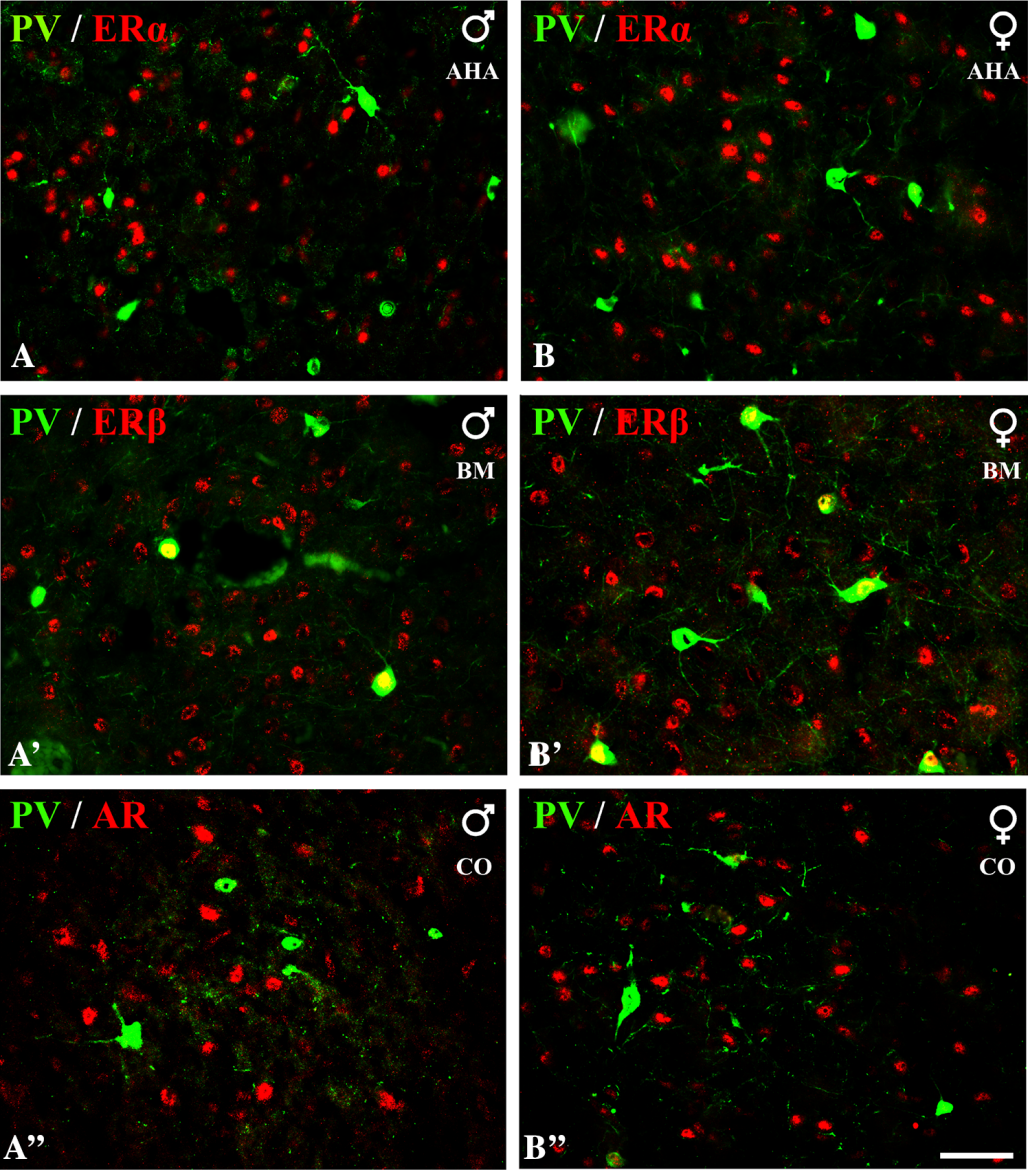
Representative colour photomicrographs illustrating the anatomical relationships between PV+ neurons and nuclei immunoreactive for ERα (**A**, **B)**, ERβ (**A’**, **B’**) or AR (**A”**, **B”**) in the amygdala of male (**A**, **A’**, **A”**) and female (**B**, **B’**, **B”**) guinea pigs. Note, extensive co-localization of PV+ neurons with ERβ (**A’**, **B’**) but not ERα (**A**, **B)** or AR (**A”**, **B”**). *AHA* amygdalo-hippocampal area, *BM* basomedial nucleus, *CO* Cortical nucleus. *Scale bar* 50 µm in **B”** (applies to all sections)

**Fig. 6:**
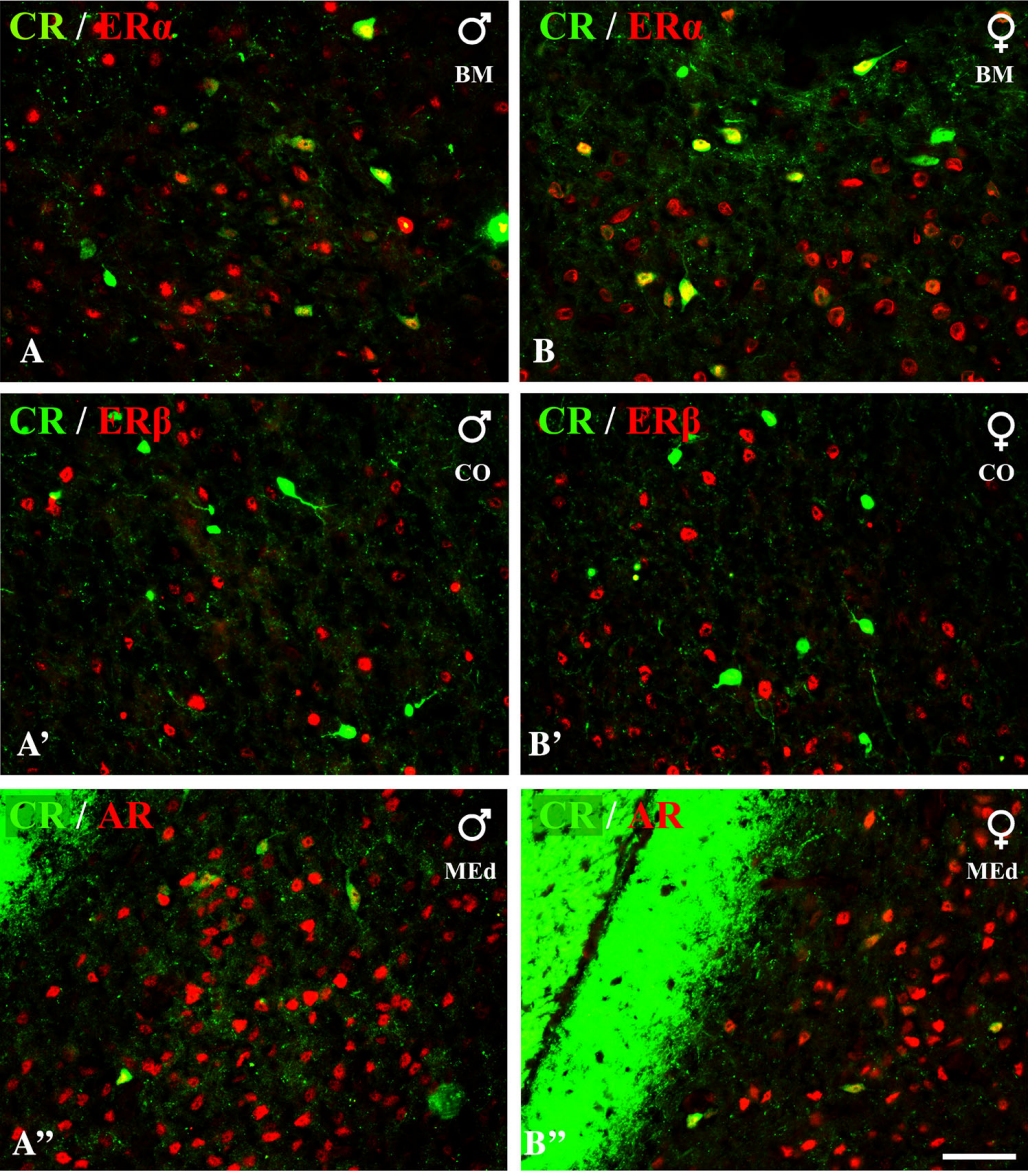
Representative colour photomicrographs illustrating the anatomical relationships between CR+ neurons and nuclei immunoreactive for ERα (**A**, **B)**, ERβ (**A’**, **B’**) or AR (**A”**, **B”**) in the amygdala of male (**A**, **A’**, **A”**) and female (**B**, **B’**, **B”**) guinea pigs. Note, extensive co-localization of CR+ neurons with ERα (**A**, **B**), but not ERβ (**A’**, **B’**). Note, also that some CR+ neurons were simultaneously immunonoreactive for AR (**A”**, **B”**). *BM* basomedial nucleus, *CO* cortical nucleus, *MEd* dorsal part of medial nucleus. *Scale bar* 50 µm in **B”** (applies to all sections)

All details concerning the co-existence of CB+, PV+ and CR+ neurons with ERα+, ERβ+ and AR+ in the amygdala of male and female guinea pig are shown in Tables 2, 3, 4 and 5 and Figs. 3, 4, 5 and 6. These details clearly demonstrate that: CB+, PV+ and CR+ neurons have different and unique patterns of co-expression with ERβ, ERα and AR; there is cellular separation of ERβ, ERα and AR within non-overlapping subpopulations of CB+ PV+ and AR+ cells; the patterns and extents of co-expression are very similar in both sexes.

**Table 2:** The co-localization pattern of CB+ neurons with ERα, ERβ and AR in the amygdala of the guinea pig in male and female subjects

	Calbindin										
	ERα			ERβ			AR				
	CB+	CB+/ERα+	%	CB+	CB+/ERβ+	%	CB+	CB+/AR	%		
LA											
♂	527.3 ± 33.7	4.8 ± 0.3	0.91 ± 0.07	564.2 ± 26.1	34.6 ± 2.4	6.13 ± 0.45	571.8 ± 43.1	0	–		
♀	560.9 ± 42.8	4.1 ± 0.4	0.73 ± 0.05	587.4 ± 47.9	31.3 ± 2.9	5.33 ± 0.31*	601.6 ± 31.7	0	–		
BL											
♂	593.5 ± 31.9	0	–	549.6 ± 28.6	18.5 ± 2.1	3.36 ± 0.22	535.8 ± 23.6	0	–		
♀	624.1 ± 50.3	0	–	605.5 ± 33.9*	24.3 ± 1.7**	4.01 ± 0.39*	574.1 ± 38.3	0	–		
BM											
♂	379.1 ± 17.4	3.2 ± 0.3	0.84 ± 0.05	432.3 ± 32.8	61.8 ± 3.6	14.29 ± 0.59	411.2 ± 27.3	23.5 ± 1.1	5.73 ± 0.55		
♀	415.5 ± 25.6	3.8 ± 0.2	0.90 ± 0.07	488.8 ± 29.5*	65.6 ± 4.3	13.43 ± 0.91	457.6 ± 35.6*	22.6 ± 1.4	4.94 ± 0.34		
CE											
♂	317.8 ± 15.9	0	–	292.8 ± 15.7	3.1 ± 0.4	1.05 ± 0.11	273.7 ± 20.2	17.2 ± 1.2	6.28 ± 0.46		
♀	308.3 ± 22.1	0	–	303.1 ± 19.3	2.6 ± 0.3	0.86 ± 0.09*	267.3 ± 15.7	19.7 ± 1.5*	7.11 ± 0.58		
ME											
♂	618.1 ± 38.2	3.1 ± 0.5	0.51 ± 0.07	650.4 ± 39.2	4.4 ± 0.3	0.68 ± 0.08	664.6 ± 29.1	241.5 ± 19.3	36.34 ± 2.45		
♀	549.8 ± 26.3	3.8 ± 0.4	0.69 ± 0.04	581.9 ± 27.8*	5.1 ± 0.4*	0.87 ± 0.11*	603.2 ± 40.2*	209.1 ± 17.1*	34.65 ± 1.89		
CO											
♂	475.7 ± 57.9	3.5 ± 0.4	0.72 ± 0.04	508.6 ± 24.1	56.2 ± 4.3	11.04 ± 0.93	518.9 ± 34.3	24.4 ± 2.1	4.71 ± 0.39		
♀	469.7 ± 42.6	2.2 ± 0.1	0.47 ± 0.06	491.9 ± 41.2	47.9 ± 3.8*	9.73 ± 0.71	487.3 ± 27.6	17.3 ± 1.3**	3.56 ± 0.43**		

Note in both sexes modest co-localization of CB+ neurons with ERβ and AR as well as the lack of associations of these cells with ERα. Data were expressed as mean ± standard deviation (SD). As CB+ neurons almost never co-express ERα, only the counts concerning relationships of CB+ neurons with ERβ or AR were statistically evaluated by the two-tailed *t* test (* *p* ≤ 0.05, ** *p* ≤ 0.01 and *** *p* ≤ 0.001)

**Table 3:** The co-localization pattern of CB+ neurons with PV or CR and SOM+ neurons with AR or ERβ in the amygdala of the guinea pig in male and female subjects

	Calbindin						Somatostatin					
	PV			PV			AR			ERβ		
	CB+	CB+/PV+	%	CB+	CB+/CR+	%	SOM+	SOM+/AR+	%	SOM+	SOM+/ERβ	%
LA												
♂	263.7 ± 20.6	72.6 ± 5.1	27.5 ± 1.94	255.8 ± 17.2	2.3 ± 0.1	0.9 ± 0.12	133.4 ± 7.7	0	–	142.2 ± 9.3	0	–
♀	279.0 ± 22.3	81.2 ± 6.7	29.1 ± 2.67	247.5 ± 16.4	1.6 ± 0.2	0.7 ± 0.06	138.7 ± 8.9	0	–	129.1 ± 8.6	0	–
BL												
♂	248.1 ± 15.7	98.5 ± 8.4	39.7 ± 3.09	232.8 ± 20.5	1.9 ± 0.2	0.8 ± 0.07	88.5 ± 6.1	0	–	95.7 ± 5.3	0	–
♀	261.3 ± 19.2	94.8 ± 7.8	36.3 ± 2.98	243.5 ± 18.6	1.3 ± 0.1	0.5 ± 0.05	97.6 ± 7.5	0	–	90.6 ± 7.2	0	–
BM												
♂	178.9 ± 12.4	63.7 ± 4.3	35.6 ± 2.72	161.6 ± 12.7	0.7 ± 0.1	0.4 ± 0.03	75.1 ± 6.2	4.6 ± 0.4	5.9 ± 0.52	65.9 ± 5.9	0	–
♀	198.2 ± 15.1	68.3 ± 5.2	34.5 ± 2.57	176.3 ± 10.8	1.1 ± 0.1	0.6 ± 0.08	86.0 ± 7.2	5.7 ± 0.6	6.7 ± 0.48	73.1 ± 4.8	0	–
CE												
♂	133.4 ± 11.5	0	–	137.9 ± 9.8	0	–	73.7 ± 4.7	6.1 ± 0.4	8.3 ± 0.71	76.4 ± 4.2	0	–
♀	126.5 ± 9.6	0	–	148.3 ± 10.3	0	–	82.4 ± 6.2	5.8 ± 0.5	7.1 ± 0.83	69.5 ± 6.7	0	–
ME												
♂	284.1 ± 21.3	0	–	264.6 ± 22.7	2.2 ± 0.3	0.8 ± 0.07	119.3 ± 8.3	46.9 ± 3.7	39.3 ± 2.89	114.1 ± 9.2	0	–
♀	267.3 ± 19.7	0	–	253.9 ± 18.1	3.1 ± 0.2	1.2 ± 0.15	107.5 ± 9.1	39.5 ± 3.4	36.6 ± 3.45	105.2 ± 7.8	0	–
CO												
♂	232.2 ± 17.2	73.1 ± 4.6	31.4 ± 2.88	208.5 ± 13.6	0	–	85.8 ± 5.4	4.9 ± 0.6	5.7 ± 0.43	79.6 ± 6.4	0	–
♀	224.8 ± 14.9	64.6 ± 5.4	28.7 ± 2.31	226.9 ± 18.4	0	–	81.9 ± 4.9	3.7 ± 0.3	4.6 ± 0.29	88.3 ± 5.1	0	–

Note in both sexes extensive co-localization of CB+ neurons with PV and the lack of co-expression of these cells with CR. Note also extensive co-localization of SOM+ neurons with AR and the lack of assotiations of these cells with ERβ. Data were expressed as mean ± standard deviation (SD). As CB+ neurons almost never co-express CR and SOM+ neurons are devoid of ERβ only the counts concerning relationships of CB+ neurons with PV and SOM+ cells with AR were statistically evaluated by the two-tailed *t* test (* *p* ≤ 0.05, ** *p* ≤ 0.01 and *** *p* ≤ 0.001)

**Table 4:** The co-localization pattern of PV+ neurons with ERα, ERβ and AR in the amygdala of the guinea pig in male and female subjects

	Parvalbumin								
	ERα			ERβ			AR		
	PV+	PV+/ERα+	%	PV+	PV+/ERβ+	%	PV+	PV+/AR	%
LA									
♂	619.3 ± 51.2	4.1 ± 0.4	0.66 ± 0.05	662.4 ± 32.3	92.3 ± 6.9	13.93 ± 0.81	628.3 ± 39.6	0	–
♀	675.6 ± 39.1	3.3 ± 0.3	0.49 ± 0.07	746.5 ± 47.9*	127.8 ± 9.8**	17.12 ± 1.42**	694.8 ± 53.5	0	–
BL									
♂	446.2 ± 27.9	0	–	467.2 ± 42.7	32.1 ± 2.2	6.87 ± 0.54	484.2 ± 35.8	0	–
♀	499.3 ± 40.8	0	–	522.8 ± 29.9	29.5 ± 2.6	5.65 ± 0.47*	537.1 ± 33.1	0	–
BM									
♂	278.9 ± 19.7	2.6 ± 0.3	0.92 ± 0.17	304.8 ± 23.1	88.7 ± 6.3	29.15 ± 2.52	316.8 ± 24.0	2.6 ± 0.3	0.81 ± 0.09
♀	301.3 ± 18.6	3.1 ± 0.4	1.02 ± 0.23	346.6 ± 15.7*	109.3 ± 7.5**	31.54 ± 1.87	332.4 ± 19.1	1.2 ± 0.1	0.34 ± 0.05
CE									
♂	9.3 ± 0.3	0	–	9.6 ± 0.8	0	–	11.2 ± 1.4	0	–
♀	7.1 ± 0.5	0	–	8.7 ± 0.7	0	–	6.1 ± 0.6	0	–
ME									
♂	15.2 ± 0.8	0	–	16.8 ± 1.3	0	–	13.3 ± 0.8	0	–
♀	13.5 ± 0.9	0	–	15.5 ± 1.1	0	–	10.6 ± 0.5	0	–
CO									
♂	237.4 ± 10.2	2.4 ± 0.2	1.03 ± 0.15	250.4 ± 19.8	65.4 ± 3.4	26.12 ± 1.27	268.1 ± 14.3	2.9 ± 0.2	1.07 ± 0.08
♀	225.2 ± 11.6	1.2 ± 0.1	0.53 ± 0.06	219.6 ± 15.9*	56.2 ± 3.8*	25.61 ± 1.43	241.9 ± 17.6	1.8 ± 0.1	0.76 ± 0.04

Note in both sexes extensive co-localization of PV+ neurons with ERβ and the lack of associations of these cells with ERα or AR. Data were expressed as mean ± standard deviation (SD). As PV+ neurons almost never co-express ERα or AR, only the counts concerning relationships of PV+ neurons with ERβ were statistically evaluated by the two-tailed *t* test (* *p* ≤ 0.05, ** *p* ≤ 0.01 and *** *p* ≤ 0.001)

**Table 5:** The co-localization pattern of CR+ neurons with ERα, ERβ and AR in the amygdala of the guinea pig in male and female subjects

	Calretinin								
	ERα			ERβ			AR		
	CR+	CR+/ERα+	%	CR+	CR+/ERβ+	%	CR+	CR+/AR	%
LA									
♂	1281.6 ± 68.8	238.4 ± 18.2	18.6 ± 0.96	1309.1 ± 96.7	0	–	1231.0 ± 76.3	0	–
♀	1317.5 ± 93.3	280.6 ± 24.7*	21.3 ± 1.45*	1328.4 ± 81.9	0	–	1198.3 ± 109.8	0	–
BL									
♂	440.9 ± 18.6	27.8 ± 2.3	6.3 ± 0.84	461.5 ± 23.0	0	–	429.4 ± 29.9	3.4 ± 0.4	0.8 ± 0.06
♀	459.4 ± 26.5	21.1 ± 2.8*	4.6 ± 0.69*	470.2 ± 27.2	0	–	442.2 ± 25	3.1 ± 0.3	0.7 ± 0.11
BM									
♂	357.7 ± 27.1	120.5 ± 9.1	33.7 ± 1.97	317.1 ± 17.7	3.5 ± 0.3	1.1 ± 0.14	334.3 ± 26.1	25.7 ± 1.8	7.7 ± 0.63
♀	369.2 ± 18.4	129.9 ± 8.2	35.2 ± 1.81	335.2 ± 24.3	2.7 ± 0.2	0.8 ± 0.09	350.3 ± 21.5	23.8 ± 1.7	6.8 ± 0.41
CE									
♂	12.3 ± 1.0	0	–	8.6 ± 0.9	0	–	11.2 ± 0.9	0	–
♀	13.6 ± 0.8	0	–	12.7 ± 1.1	0	–	12.7 ± 0.7*	0	–
ME									
♂	422.6 ± 25.2	127.2 ± 6.6	30.1 ± 1.24	362.3 ± 23.9	2.8 ± 0.1	0.8 ± 0.05	394.9 ± 31.6	70.3 ± 5.1	17.8 ± 1.13
♀	403.8 ± 37.1	112.7 ± 8.6*	27.9 ± 2.01	349.0 ± 14.5	1.7 ± 0.1	0.4 ± 0.03	377.6 ± 28.1	57.8 ± 4.2**	15.3 ± 0.85*
CO									
♂	386.4 ± 23.9	90.4 ± 3.9	23.4 ± 1.76	396.4 ± 28.4	0	–	361.3 ± 19.2	13.0 ± 0.6	3.6 ± 0.28
♀	370.7 ± 17.8	84.5 ± 5.8	22.8 ± 1.36	407.6 ± 21.5	0	–	339.7 ± 25.1	13.9 ± 0.8	4.1 ± 0.37

Note that in both sexes CR+ neurons show extensive co-localization with ERα and modest co-expression with AR, however, these cells almost never co-express ERβ. Data were expressed as a mean ± standard deviation (SD). As CR+ neurons almost never co-express ERβ, only the counts concerning relationships of CR+ neuron with ERα or AR were statistically evaluated by the two-tailed *t* test (* *p* ≤ 0.05, ** *p* ≤ 0.01 and *** *p* ≤ 0.001)

### Calbindin neurons

Small proportions of CB+ neurons in the amygdala were costained for ERβ or AR, but these cells almost never co-expressed ERα (Table 2; Fig. 3). Double-labelled neurons for CB and ERβ were observed in all the nuclei studied, although the percentages differed substantially among various regions. For example, within the lateral and basolateral nuclei, less than 7% of CB+ neurons co-expressed ERβ, but within the basomedial nucleus, the percentage of such neurons was two times higher. Within the central and medial nuclei, double-labelled cells were observed only occasionally, while within the cortical nuclei approximately 10% of CB+ cells were costained for ERβ. Double-labelled neurons for CB and AR were observed within the central, medial, basomedial and cortical nuclei (Table 2; Fig. 3). With the exception of the medial nucleus, where almost 37% of CB+ neurons were AR+, in the other regions such double-labelled cells were much less numerous.

As population of CB+ neurons in the amygdala consists of two separate and non-overlapping subpopulations such as CB+/PV+ and CB+/SOM+ cells, additional staining was performed to verify which CB+ neurons may co-express ERβ or AR (Fig. 4). Double-immunofluorescence with antibodies against CB and PV revealed the substantial overlap of both markers, suggesting that at least a part of CB+/ERβ+ neurons may contain PV as well (Fig. 4a). For example, in the lateral and basolateral nuclei, approximately 30 and 40% of CB+ neurons co-expressed PV in male and female subjects, respectively (Table 3). Double-immunofluorescence with antibodies against SOM and ERβ demonstrated the lack of any co-existence of both these markers in the same neurons (Table 3; Fig. 4c). In contrast, SOM and AR co-existed in the same cells in the medial nucleus and neighbouring regions (Fig. 4d–f). In the medial nucleus approximately 40% of SOM+ neurons were simultaneously AR+ (Table 3). In the basomedial, cortical and central nuclei, only single cells were double-labelled for SOM and AR (Table 3).

### Parvalbumin neurons

A substantial proportion of PV+ neurons in the amygdala co-expressed ERβ, but these cells almost never co-expressed ERα or AR (Table 4; Fig. 5). Within the lateral and basolateral nuclei, approximately 15 and 7% of PV+ neurons co-expressed ERβ, respectively, whereas within the basomedial nucleus one-third of these cells were simultaneously ERβ+. Within the medial and central nuclei, there were no neurons double-labelled for PV and ERβ due to the limited number of PV+ cells in both these regions. Within the cortical nuclei, double-labelled cells were rarely observed in more anterior portions of the nucleus. However, approximately one-third of PV+ cells co-expressed ERβ within the periamygdaloid cortex and posterior cortical nucleus.

### Calretinin neurons

In contrast to CB+ and PV+ neurons, CR+ cells often co-expressed ERα, but these cells almost never co-expressed ERβ (Table 5; Fig. 6). Moreover, across diverse regions of the amygdala, there were also dispersed CR+ neurons immunoreactive to AR (Table 5; Fig. 6). Within the lateral nucleus, approximately 20% of CR+ neurons co-expressed ERα. Within the basolateral, nucleus the density of ERα+ cells was low, so the percentage of CR+ neurons co-expressing ERα was one-third of that in the lateral nucleus. Within the basomedial and medial nuclei more than one-third of CR+ neurons co-expressed ERα, and one quarter of such CR+/ERα+ cells were observed within cortical nuclei. Double-labelled neurons for CR and AR were observed within the medial, basomedial and cortical nuclei (Table 5; Fig. 6). In the medial and basomedial nuclei approximately 16 and 7% of CR+ neurons co-expressed AR, respectively. In the cortical nuclei less than 5% of CR+ cells were AR+.

Statistical evaluation of numbers of cells expressing various calcium-binding proteins, cells double-labelled for both calcium-binding proteins and steroid receptors, as well as percentages of double-labelled cells indicated the sex diversified sensitivity of various amygdala regions to sex hormones. For example, the lateral and basomedial nuclei are mostly controlled by oestrogens via PV+ neurons using ERβ and CR+ cells using ERα. Such impact is stronger in females (Tables 4, 5). On the other hand, the medial nucleus is mostly controlled by androgens via CB+ and CR+ neurons using AR and by oestrogens via CR+ neurons using ERα. In both these cases, such influence is stronger in males (Tables 2, 5).

## Discussion

To the best of our knowledge, the present study describes for the first time a systematic analysis of relationships between populations of CB+, PV+ and CR+ neurons, which form in the amygdala main subsets of GABA+ system and neurons bearing steroid receptors such as ERα or ERβ or AR. The results show that in the amygdala of male and female guinea pig, subpopulations of the CB+ and PV+ neurons co-express ERβ, while many of the CR+ neurons co-express ERα. In addition, in the medial nucleus and some other neighbouring regions there are also subpopulations of CB+ and CR+ neurons which co-express AR. The localization of ERα, ERβ or AR within subsets of GABAergic interneurons across diverse amygdaloid regions suggests that steroid hormones may exert a significant influence over local neuronal activity by directly modulating inhibitory tone. The control of inhibitory tone may be a mechanism whereby oestrogen and androgen modulate amygdalar processing in a sex-specific manner. Moreover, as the numbers of CB+, PV+ and CR+ neurons (Równiak et al. 2015) and the numbers of such cells co-expressing steroid receptors (present results) are sexually dimorphic in various nuclei of the guinea pig amygdala, sensitivity to sex hormones and their impact on many regions may substantially differ in male and female subjects. Thus, GABA+ neurons co-expressing CB+, PV+ or CR+ provide a precisely designed inhibitory interface which is steroid-sensitive and sexually dimorphic. This interface could be the natural basis for sex-specific processing in the amygdala.

The present results demonstrate that in the amygdala of the guinea pig, subpopulations of CB+, PV+ and CR+ neurons are oestrogen or androgen-sensitive as many of these cells co-express ERα, ERβ or AR. The patterns of co-expression are similar in both sexes. However, these patterns are quite different in the “cortex-like” nuclei (the cortical and basolateral amygdala) and “striatum-pallidum-like” nuclei (the central and medial amygdala). In the “cortex-like” regions, CB+, PV+ and CR+ neurons use mostly oestrogen receptors, and the pattern of co-expression is similar to relationships observed in the cerebral cortex (Blurton-Jones and Tuszynski 2002; Kritzer 2002). In the “striatum-pallidum-like” nuclei PV+ neurons are not present, while CB+ and CR+ cells are much more influenced by androgens. However, there are no data for comparisons in the available literature. As the amygdala is composed of “cortex-like” and “striatum-pallidum-like” nuclei (Swanson and Petrovich 1998), one important fact should be kept in mind. In the “cortex-like” regions, most projection neurons (pyramidal and semi-pyramidal cells) use glutamate as a fast, excitatory neurotransmitter (McDonald 1996), whereas, in contrast, many projection cells of the “striatum-pallidum-like” nuclei use GABA as a fast, inhibitory neurotransmitter (Swanson and Petrovich 1998; Saha et al. 2000; Seo et al. 2016). In the “cortex-like” regions, GAD is expressed in interneurons (McDonald 1985; Pitkänen and Amaral 1994), whereas in the “striatum-pallidum-like” nuclei, it is often expressed in projection neurons (Swanson and Petrovich 1998; Saha et al. 2000; Seo et al. 2016). The finding that substantial subpopulations of PV+ neurons and small subsets of CB+ neurons in the amygdala of the guinea pig co-express ERβ, while these cells almost never co-express ERα coincide well with some previous studies on the “cortex-like” amygdala (Blurton-Jones and Tuszynski 2002; Perez et al. 2004) and cerebral cortex (Kritzer 2002) of the rat. For example, extensive co-localization of PV+ neurons with ERβ was reported in this species in the lateral and basomedial nuclei of the amygdala (Blurton-Jones and Tuszynski 2002) and sensorimotor and association areas of the cerebral cortex (Blurton-Jones and Tuszynski 2002; Kritzer 2002). The co-expression of CB+ neurons with ERβ in the sensorimotor and association cortices was rather limited. However, in the piriform and enthorinal cortices, such co-expression was much more extensive (Kritzer 2002). On the other hand, there is no or very weak co-expression between CB+ or PV+ neurons with ERα in the amygdala (present results; Perez et al. 2004) and various areas of the cerebral cortex (Perez et al. 2004; Kritzer 2002). The present results also demonstrate that substantial populations of CR+ neurons in the amygdala of the guinea pig co-express ERα, while these cells were almost without exception immunonegative for ERβ. These findings are in agreement with observations in the juvenile rat cerebral cortex, which revealed substantial overlap between CR+ and ERα+ immunoreactivity but virtually no co-localization of CR+ and ERβ+ immunolabelling (Hayashi et al. 2001; Kritzer 2005). The cells double-labelled for CR and ERα were also described in the adult rat hippocampus (Nakamura and McEwen 2005). Finally, the present results demonstrate that in the medial nucleus and in some other neighbouring regions, there are also subpopulations of CB+ and CR+ neurons which co-express AR. Data concerning relationships between CB+ neurons and AR in the amygdala are not as yet available. In the cerebral cortex AR is mostly present in the pyramidal neurons, and in the most cortical areas virtually none of the non-pyramidal CB+ or PV+ neurons were found to be immunoreactive for AR (Kritzer 2004). Only in the proisocortical areas of the piriform and entorhinal cortices, many of the non-pyramidal CB+ or PV+ cells were simultaneously AR+ (Kritzer 2004). Data concerning relationships between CR+ neurons and AR in the amygdala or cerebral cortex are not yet available.

The present results demonstrate that there is strict cellular separation of ERβ in CB+ and/or PV+ neurons and ERα in CR+ cells. In contrast, AR is expressed in non-overlapping subpopulations of CB+ and CR+ cells, because CR and CB were not co-localized in amygdala neurons of the guinea pig. Such separaration of ERα, ERβ or AR within subsets of non-overlapping subpopulations of GABA+ interneurons does not seem to be accidental, and this suggests that these steroid-sensitive subsets form a kind of precisely designed interface for sex hormones’ actions. For example, there is no or very little overlap in adult life between immunoreactivity for ERβ and ERα in any major region of the cortex, hippocampus or amygdala (Kritzer 2002; Perez et al. 2004), although there are brain regions where such co-existence exists (Gréco et al. 2001). There is also no or very little overlap between CR+ cells either with CB+ or PV+ neurons in the cortex, hippocampus or amygdala (present results, and Miettinen et al. 1992; Mascagni et al. 2009). However, in all these areas there is extensive co-localization of CB and PV in the same neurons (present results, and Mascagni et al. 2009). Moreover, there are intriguing parallels in the development of ERβ+ and PV+ neurons on the one hand and ERα+ and CR+ cells on the other, suggesting that the cellular separation observed in adult life is established during brain development (Kritzer 2005). Specifically, in the postnatal rat cerebrum, like ERβ+ cells, PV+ neurons are identified initially in low numbers around PND 7 (7th day of postnatal life), and from that time steadily increase in number and density to reach mature patterns of labelling by the end of the third postnatal week (Alcántara et al. 1993; Solbach and Celio 1991; Sánchez et al. 1992; Hof et al. 1999). In contrast, like ERα+ cells, CR+ neurons peak in density early, at about PND 7, transiently include large numbers of pyramidal and non-pyramidal cells, and sharply decrease from peak densities in early postnatal life to much lower, near-adult values by PND 15–21 (Fonseca et al. 1995; Schierle et al. 1997; Hof et al. 1999). Finally, double-labelling studies in the rat and monkey indicated that in the adult mammalian amygdala, there are at least three non-overlapping subpopulations of GABA+ interneurons: (1) PV+ neurons (most of which also contain CB); (2) SOM+ neurons (most of which also contain CB and/or neuropeptide Y); and (3) CR+ neurons (most of which also contain cholecystokinin and/or vasoactive intestinal polypeptide) (McDonald and Mascagni 2001, 2002; Mascagni and McDonald 2003; Mascagni et al. 2009). Interestingly, the present results indicate that the first population uses ERβ to respond to sex hormones. The second one is endowed with AR. The third subpopulation co-expresses usually ERα, but some of these neurons also utilize AR. It is worth mentioning that the present work was done in the guinea pig, which could be a good model system for studying brain sexual dimorphism, as well as neuroendocrine and developmental processes because of the many similarities to primates and humans (Wallen and Baum 2002; Bartesaghi and Severi 2004, Kaiser and Sachser 2005). As the results in the guinea pig are quite similar to available data obtained from the rat (Blurton-Jones and Tuszynski 2002) and monkey (Perez et al. 2004), it seems that in the amygdala the co-expression patterns of neurons containing calcium-binding proteins and steroid receptors may be similar across various mammalian species.

The present results seem to be very interesting in the light of synaptic studies which indicated that PV+, CB+ and CR+ neurons perform quite different roles in the inhibitory mechanism of the amygdala (Muller et al. 2007; Miles et al. 1996; Woodruff and Sah 2007a). It should be added, however, that available data are limited to the “cortex-like” amygdala only. For example, PV+ neurons are considered to be a critical component of the inhibitory circuitry in the basolateral and cortical amygdala, where they form characteristic basket-like plexuses around the unstained somata and cartridges, which represent axo-axonic contacts on initial segments of axons (McDonald and Mascagni 2001; Muller et al. 2005; Woodruff and Sah 2007b). Thus, as in the cortex, many PV+ interneurons in both the rodent and primate “cortex-like” amygdala appear to be basket or chandelier cells that provide a strong perisomatic inhibition of local pyramidal neurons (Pitkänen and Amaral 1993; Sorvari et al. 1995, 1996; Woodruff and Sah 2007a, b). Such inhibition is considered to control the output, most notably the synchrony of action potentials of large principal cell populations (Cobb et al. 1995; Miles et al. 1996). Taking into account that many PV+ neurons in the amygdala co-express ERβ (present results, and Blurton-Jones and Tuszynski 2002), and that the numbers of these cells in various amygdaloid nuclei are sexually dimorphic (Równiak et al. 2015; Stefanova et al. 1997), the mechanism described above may be strongly modulated by circulating steroid hormones and sex-specific. CB+ neurons perform a dual role in the intrinsic inhibitory mechanism of the amygdala. In rodents (but not primates) some of them appear to be basket cells, which provide a strong perisomatic inhibition of local pyramidal neurons, and such cells co-express PV but do not contain somatostatin and/or neuropeptide Y (Pitkänen and Amaral 1993; Sorvari et al. 1995, 1996; Woodruff and Sah 2007a, b). The other CB+ neurons are considered as dendrite-targeting interneurons, providing a robust innervation of dendrites in the rat and primate amygdala (Mascagni et al. 2009). Such neurons contain somatostatin and/or neuropeptide Y (but not PV) (McDonald and Mascagni 2002; Muller et al. 2007), and are responsible for the control of the efficacy and plasticity of inputs from specific sources that terminate in the same dendritic domain (Cobb et al. 1995; Miles et al. 1996). Interestingly, it seems probable that the double-labelled CB+/ERβ+ neurons observed in the present study are those CB+ cells which contain PV. On the other hand, CB+/AR+ neurons are most probably those CB+ cells which co-express somatostatin. Thus, both subpopulations of CB+ neurons and both mechanisms described above may be modulated by circulating steroid hormones and sex-specific. There are no detailed synaptic studies on the amygdala concerned with CR+ neurons. However, such studies in the neocortex revealed that CR+ neurons are mostly dendrite-targeting, and their buttons typically innervate dendritic shafts, and less often dendritic spines or somata (Meskenaite 1997). Moreover, at least in some cortical areas and layers, CR+ neurons innervate preferentially other GABA+ neurons (Melchitzky and Lewis 2008), and by inhibiting other classes of inhibitory interneurons they may exert a significant disinhibitory effect on the pyramidal neurons (Meskenaite 1997; Gonchar and Burkhalter 1999). Analogous relationships were also described in the hippocampus, where CR+ interneurons were found to be specialized to control other interneuronal types (Gulyás et al. 1996). Taking into account that many CR+ neurons in the amygdala co-express ERα or AR (present results), the mechanisms described above may be strongly modulated by both oestrogen or testosterone, and thus sex-specific.

The present results and some other studies (Krȩżel et al. 2001; Tan et al. 2012; Blurton-Jones and Tuszynski 2002) provide evidence that there is a relationship between steroid hormone receptors and inhibitory tone in the amygdala. Thus, the question arises what may be the functional significance of such a relationship. There are several lines of evidence that modulation of inhibitory tone by oestrogen and/or androgen via steroid receptors on GABAergic cells may be important for proper amygdala physiology, and any alterations in such modulation may lead to various psychiatric disorders, which differentially affect men and women (prevalence, severity) (Krȩżel et al. 2001; Kinrys and Wygant 2005). For example, studies in ERβ knock-out mice revealed that mutant females displayed increased anxiety and/or depression-like behaviour associated with a reduced threshold for the induction of synaptic plasticity in the basolateral amygdala (Krȩżel et al. 2001). A reduced threshold in this structure could result in exaggerated responses to normally innocuous stimuli or environments, a characteristic feature of fear and anxiety disorders. Whether a reduction of GABA responses in the basolateral amygdala is the mechanism by which anxiety is increased in ERβ mutant mice remains to be tested, but some evidence indicates that pharmacological manipulation of GABA type A receptors in wild type mice can mimic some of the effects of ERβ null mutation (Krȩżel et al. 2001). Moreover, selective ERβ agonists typically exert potent anxiolytic activity (Lund et al. 2005; Oyola et al. 2012). Such anxiolytic effects are mediated by the cortex and hippocampus, and most probably also by the basolateral amygdala, by elevation of GABAergic inhibitory signalling and reduction of glutamatergic excitatory drive (Tan et al. 2012). On the other hand, male ERα knock-out mice display reduced aggression (Ogawa et al. 1997, 1998), while selective ERα agonists typically elevate anxiety and/or aggression (Nomura et al. 2002). All these data from ERβ and ERα knock-out mice are in accordance with recent physiological studies (Adhikari et al. 2015; Hong et al. 2014) and the present description of ERα, ERβ and/or AR enrichment in several amygdala nuclei. For example, the basomedial nucleus is the region in the amygdala, which is directly engaged in anxiolysis, however, the medial and cortical nuclei cannot be excluded as well (Adhikari et al. 2015). Interestingly, in all these nuclei, ERβ is very abundant according to present results. On the other hand, in the medial nucleus, which is especially rich in ERα and other steroid receptors, there are many aggression-promoting GABAergic neurons (Hong et al. 2014).

## Conclusions

The results of the present study provide evidence that in the amygdala of the guinea pig subpopulations of CB+ and PV+ neurons co-express ERβ, while many of CR+ neurons co-express ERα. In addition, in the selected nuclei, there are also subpopulations of CB+ and CR+ neurons which co-express AR. The localization of ERα, ERβ or AR within subsets of GABAergic interneurons across diverse amygdaloid regions suggests that steroid hormones may exert a significant influence over local neuronal activity by directly modulating inhibitory tone. The control of inhibitory tone may be one of the mechanisms whereby oestrogen and androgen could modulate amygdala processing in a sex-specific manner. Another mechanism may be thorough steroid-sensitive projection neurons, which are most probably located in the medial and central nuclei.
